# The Indiana Dental Law

**Published:** 1879-07

**Authors:** 


### ARTICLE VIII.

### *Indiana Dental Law.*

The following is a copy of the law regulating the practice of dentistry in the State of Indiana, enacted by the Legislature of that State at its recent session.

SEC. 1. *Be it enacted, by the General Assembly of the State of Indiana,* That it shall be unlawful for any one to practice dentistry for a fee or reward, in the State of Indiana, without having received a diploma from a Dental College, duly incorporated under the laws of this or some other State of the United States, or a certificate of qualification, issued by a Board of Examiners to be appointed by the Indiana State Dental Association: *Provided*, That nothing in this act shall apply to any one engaged in the practice of dentistry in this State at the time of the passage of this act.

SEC. 2. A Board of Examiners, consisting of five practicing dentists, shall be appointed by said State Dental Association, according to its by-laws, whose duty it shall be to meet annually at the time and place of meeting of the said State Association, or oftener, at the call of three members of said Board, at such time and place as may be designated in said call, and to examine all who pass a satisfactory examination.

SEC. 3. Any applicant who furnishes satisfactory proof of having heen engaged in the reputable practice of dentistry for ten consecutive years immediately preceding the time of their application, shall be examined only in practical dentistry—operative and mechanical; all others shall be examined in Anatomy, Physiology, Pathology, Therapeutics, Chemistry, and the theory and practice of surgical and mechanical dentistry.

SEC. 4. All certificates issued under the provisions of this act shall be signed by all the members of said Board of Examiners, and have the seal of the Indiana State Dental Association affixed, and shall be prima facie evidence of the right of the holder to practice under this act, which right it shall be incumbent on the holder to prove in all prosecutions under the same.

SEC. 5. Any member of the Board of Examiners may grant a permit to practice until the next meeting of the board, but such permit shall be valid only until said next meeting, and in no case be extended or renewed.

SEC. 6. Any person violating the provisions of this act shall be liable to prosecution, upon complaint of any citizen of this State, before a Justice of the Peace, or in any Superior Court of record, by indictment or information, in the county where the offense is committed; and upon conviction, shall be fined in any sum not less than fifty nor more than one hundred dollars for each offense: *Provided*, Nothing in this act shall be so construed as to prevent physicians or surgeons extracting teeth; and all fines so collected, shall belong to the common school fund of the county where assessed.

SEC. 7. To provide a fund to carry out and enforce the provisions of this act, the Board of Examiners shall, before examination, collect from each applicant, the sum of twenty-five dollars; any portion of which there may be remaining after paying necessary expenses attending such examination shall be paid into the treasury of the said State Association, to be used for the purpose for which said fund is hereby created.

SEC. 8. Three members of the Board of Examiners shall constitute a quorum, and all questions before them shall be decided by a vote of the majority of those present; and should there not be a quorom present on the day of meeting, those present may meet and adjourn from day to day, until there is a quorum present.

SEC. 9. The Board shall receive, out of the fund created by this act, such compensation for their services as the bylaws of said Dental Association may provide.

SEC. 10. This act shall be in force from and after its passage, publication and circulation in the several counties of the State.

---

